# Structural analysis of a fibre-reinforced composite blade for a 1 MW tidal turbine rotor under degradation of seawater

**DOI:** 10.1007/s40722-023-00279-w

**Published:** 2023-03-16

**Authors:** Yadong Jiang, William Finnegan, Finlay Wallace, Michael Flanagan, Tomas Flanagan, Jamie Goggins

**Affiliations:** 1 SFI MaREI Centre for Energy, Climate and Marine, Ryan Institute & School of Engineering, University of Galway, Galway, Ireland; 2 ÉireComposites Teo, An Choill Rua, Inverin, Co., Galway, Ireland; 3 Orbital Marine Power, Innovation Centre, Orkney, Scotland, UK; 4 Construct Innovate, University of Galway, Galway, Ireland

**Keywords:** Ocean energy, Tidal turbine rotor blade, Digital twin, Experimental testing, Finite-element analysis, Seawater degradation

## Abstract

This paper presents a structural performance study of a fibre-reinforced composite blade for a 1 MW tidal turbine rotor blade that was designed for a floating tidal turbine device. The 8-m long blade was manufactured by ÉireComposites Teo and its structural performance was experimentally evaluated under mechanical loading in the Large Structures Research Laboratory at the University of Galway. Composite coupons, applied with an accelerated ageing process, were tested to evaluate the influence of seawater ageing effects on the performance of the materials. The material strength of the composites was found to have a considerable degradation under the seawater ingress. As part of the design stage, a digital twin of the rotor blade was developed, which was a finite-element model based on layered shell elements. The finite-element model was verified to have good accuracy, with a difference of 4% found in the blade tip deflection between the physically measured test results in the laboratory and numerical prediction from the model. By updating the numerical results with the material properties under seawater ageing effects, the structural performance of the tidal turbine blade under the working environment was studied. A negative impact from seawater ingress was found on the blade stiffness, strength and fatigue life. However, the results show that the blade can withstand the maximum design load and guarantee the safe operation of the tidal turbine within its design life under the seawater ingress.

## Introduction

As one of the main alternatives to fossil fuels, renewable energy is developing rapidly in recent decades. In 2019, renewable energy contributed about 10% of the global total primary energy demand (IEA 2020). As regulated by the moon, the tidal stream is almost 100% predictable, making it a reliable renewable energy resource. Despite the multiple countrywide lock-downs due to COVID-19, there was significant growth in the sector, with the cumulative tidal stream technology deployed in Europe was 27.9 MW in 2020, making up 77% of the global total tidal energy device installations (OEE 2021). By 2030, the tidal energy devices predicted to be deployed in Europe are 1324 MW and 2388 MW under the low growth and high growth scenarios, respectively (OEE 2020). As tidal turbines convert ocean current to kinetic energy through rotor rotating, the structural performance of tidal turbine blades is a key concern. Since the water density is 835 times higher than that of air, tidal turbine blades suffer from complex loading. Hence, there are significant challenges regarding the design and performance validation of a tidal turbine blade.

Physical experimental testing can ensure blade safety under extreme tidal loading conditions. In recent years, there were many water tank tests of tidal turbines carried out, such as the bi-directional tidal turbine metal rotors tested by Liu et al. (2014), the 1/20th scale tidal turbine tested by Doman et al. (2015), the 1/10 scale tidal turbines tested by Jeffcoate et al. (2016), and the 1/20th scale tidal turbines tested by Porter et al. (2020). Besides the onsite tests, operation trials in sea sites were also performed by Li et al. (2019), where a 600-kW tidal current turbine was designed, manufactured and tested. In these tests, influences of turbine geometry, current condition, blade material and operation control on the power generation of tidal turbines are of main concern. Besides the turbine system performance, the structural behaviour of the tidal turbine blade is also important, as it guarantees the turbine normal operation throughout its design life. Accelerated life testing of a 3/8 scaled-down blade of a hubless turbine was carried out by Torre et al. (2018), where 778,000 cycles of fatigue loads were applied to the blade. Full-scale helical foils for the ORPC tidal energy device were tested under static and fatigue loads by Meier et al. (2020). After the tests, this carbon-fibre made foil was deployed in Alaska for operation. Glennon et al. (2021) performed accelerated life tests on a full-scale blade of a 70 kW tidal turbine. The applied fatigue loads were equivalent to 20 years of turbine operation.

For predicting the stiffness, strength and fatigue life, finite-element (FE) analysis is commonly performed in the structural design of tidal turbine blades. For modelling wind turbine blades, layered shell elements are usually used (Wang et al. 2016; Fagan et al. 2017a, 2018; Peeters et al. 2018; Finnegan et al. 2021b). Since the structure of tidal turbine blades is similar to that of wind turbine blades, this modelling methodology has also been utilised to design tidal turbine blades (Grogan et al. 2013; Murray et al. 2016; Fagan et al. 2016, 2019; Jiang et al. 2019). However, when it comes to tidal turbines with mega power output, there is a lack of available testing results to validate the accuracy and efficiency of the developed FE models. Moreover, due to the limitation of the test environment, some structural tests of tidal turbine blades, especially the fatigue tests, were conducted in the laboratory under dry conditions, which may not represent the working environments of the blades precisely.

This paper presents the structural behaviour study of a rotor blade for a 1 MW tidal turbine nacelle, which is one of the largest tidal turbines in the world, based on experimental testing and numerical analysis. The properties of the blade materials were characterised by testing coupons with the consideration of the impact of seawater immersion during operation. Through a series of testing programmes, including the natural frequency tests and the static tests, the preliminary structural performances of the 8-m tidal turbine blade were obtained. Along with the physical testing, a digital twin in the form of an FE model was developed for predicting the structural response of the blade. The accuracy of the FE model is validated against experimental data. By performing numerical analyses with seawater ageing effects considered, the structural behaviour of the 1 MW tidal turbine blade under the design loads, in terms of stiffness, risks of failure and fatigue life, are studied.

## Methodology

Physical testing is an effective and reliable method to ensure tidal turbine blade safety under extreme load cases. However, the blades are usually tested in the laboratory under dry conditions, which do not represent the marine environment that they operate in. Hence, the influence of seawater on the blade material strength cannot be considered. Therefore, in this study, numerical analyses are conducted for considering the seawater ageing effects. The degraded composite properties are obtained from coupon tests with an accelerated ageing process applied. The numerical analyses are performed based on an FE model, which is developed and validated against the experimental results. With the combination of degraded composite properties and verified FE model, the performance of the 1-MW tidal turbine blade under extreme load cases can be de-risked.

### Blade description

**Fig. 1:**
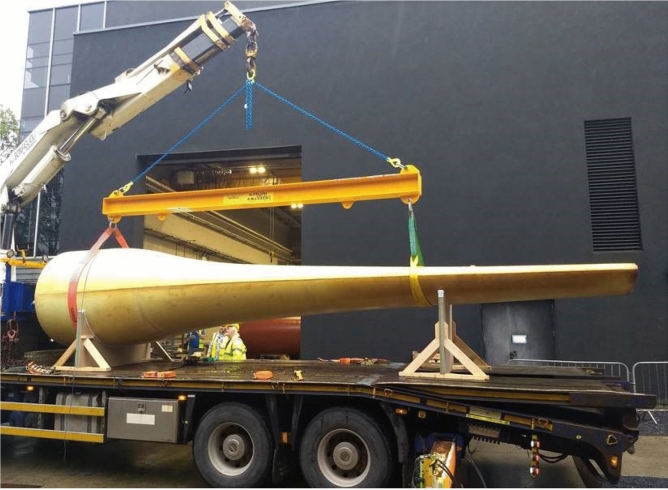
The 1-MW tidal turbine blade (manufactured at ÉireComposites)

The 8 m tidal turbine rotor blade studied in this research was designed for a floating tidal energy conversion device of Orbital Marine Power Ltd (https://orbitalmarine.com/). The tidal turbine device contains two 1 MW rotor nacelles, each with a rotor diameter of 20 m. The tidal blade consists of the main body, the trailing edge fairings and the tip. Figure 1 shows the main body of the tidal turbine blade. It was manufactured by ÉireComposites using glass fibre-reinforced powder epoxy composite material, based on the company’s Composites Powder Epoxy Technology (CPET). Further details about the manufacturing of the blade can be found in Finnegan et al. (2021a). In comparison to the conventional glass fibre-reinforced polymer (GFRP), the GFRP manufactured based on CPET has certain advantages, including small through-thickness wet out requirement, good fibre volume fraction control and low exotherm during cure. Regarding the raw material, different from traditional epoxy resins, powder epoxy can be stored at ambient temperatures and has a long shelf life. The blade trailing edge fairings and the tip are to be manufactured separately from the main body. Since the two components are to optimise the hydrodynamic shape of the blade, they are considered not to contribute significantly to resisting the primary hydrodynamic loads. Therefore, the trailing edge fairings and the tip are not included in the performance analysis.

### Material properties

The blade is mainly manufactured using uni-directional glass-fibre laminates (UD), glass-fibre woven laminates (BX) and glass-fibre triaxial laminates (TX) in conjunction with powder epoxy. The fabric used to manufacture the UD laminate has all fibre oriented at 0° while that of the BX laminate has 50% of the fibre woven at 0° and 90°, respectively. The TX laminates are manufactured using fabric with fibre uniformly woven in 0°, 60° and 120°. Further details on the characteristics of epoxy powders can be found in the research works carried out by Maguire et al. (2018, 2020). In this research, coupon tests were performed to characterise the material properties of the two composites. Given that the tidal turbine blade will be working in a marine environment for its design life, the influences of material saturation and degradation on the material properties should be considered. In the following subsections, the static tensile and fatigue testing results are given in brief. Further details can be found in the works carried out by Finnegan et al. (2021a)*.* It should be noted that in this research, only the properties of UD and BX laminates are tested, as the TX can be considered as the combination of one UD ply and one BX ply.

#### Accelerated ageing progress

The operational temperature and service life of the turbine are 12 °C and 25 years, respectively. According to the research work of Kennedy et al. (2018), the ageing procedure can be accelerated by increasing the diffusion of moisture into the polymer resin within the composite. This can be achieved by increasing the water temperature. Purnell et al. (2008) proposed the acceleration factor (*F*_*H,L*_), defined in Eq. (1), to describe the speed of acceleration progress.

1 $${F}_{H,L}={e}^{-[\frac{E}{R}(\frac{1}{{T}_{H}}-\frac{1}{{T}_{ref}})]}.$$

*E* is the activation energy, *R* is the universal gas constant (8.3145 kJ kmol^−1^ K^−1^), *T*_*H*_ is the high temperature and *T*_*ref*_ is the reference temperature (12 °C). Kennedy et al. (2018) worked out taht the activation energy of the CPET materials is 93 kJ kmol^−1^. Based on Eq. (1), it was calculated that at a temperature of 50 °C, an acceleration factor of 101 was estimated. It means that conditioning the coupons at a temperature of 50 °C for 183 days can achieve an equivalent operational time of 50 years.

To represent the operating environment of the turbine blade, an accelerated ageing process was carried out by conditioning the specimens in a heated tank of artificial seawater. The artificial seawater was produced using the ‘Seamix’ salt, sourced from Peacock Salt, Scotland. A 200-L polyethene tank, installed in an insulated wooden container, was used as the conditioning tank, with the artificial seawater maintained at 50 °C to accelerate the ageing progress of the coupons. To ensure uniformity of temperature and salinity, the water was continuously recirculated using a submerged pump, with a flow rate of approximately 2000 l/h. The equivalent operational time considered in this research are 50 years. This is more than a typical design life for a tidal turbine of 25 years. Hence, using the conditioned material data to perform the structural analysis will ensure that the results are on the safe side. The following subsections present a summary of the coupon tests. Further details about the accelerated ageing progress of the coupons can be found in Finnegan et al. (2021a)*.*

#### Coupon tests

To derive the material properties, coupon testing was performed, which involved the following four types of tests, including the standards they were performed according to:

- Tensile strength tests—ISO 527 (2009).
- Compression strength tests—ASTM D6641 (2009)
- Inter-laminar shear strength tests—ISO 14130 (1997)
- In-plane shear strength tests—ISO 14129 (1997)

All mechanical tests were conducted under room temperature and dry conditions, which are taken at 23 ± 2 °C and 50 ± 5% relative humidity. The UD coupons can be categorised into three groups, namely the un-conditioned coupons, 2-month conditioned coupons and 6-month conditioned coupons. The inter-laminar shear strength of the conditioned coupons was not tested. For the BX composites, only the unconditioned coupons were prepared and tested. The test results showed that the composite materials have a fibre volume fraction of 50%.

The testing results of the UD and BX composites are listed in Tables 1 and 2, respectively. All values expressed are mean results with the corresponding standard deviations. It is noted that the inter-laminar shear strength of the conditioned coupons was not covered in the coupon tests. In general, the material experienced performance degradation under the ageing effects of seawater, in both strength and elastic modulus. The tensile strength of UD has the highest degradation, with the strength dropping 14% and 35% under the 2-month and 6-month conditioned cases, respectively. This high strength reduction is due to the change of failure modes. For unconditioned coupons, the failure mode is dominated by fibre fracture. However, for conditioned coupons, the failure mode is a combination of fibre fracture and delamination since matrix is more sensitive to moisture compared to fibre. This results in a significant strength degradation. The UD’s 90° compression modulus and in-plane shear modulus have a relatively low degradation (2% ~ 5%) under the 6-month conditioned case. It is also observed that the 0° compression modulus of the UD has an increase of 3% and 2% under the 2-month and 6-month conditioned cases. For the other properties of UD composites, the degradation ranges from 7 to 14%. From the testing of coupons under different condition levels, it can be concluded that the seawater ingress will generally have a negative influence on the material properties. If not considering this degradation in the design stage of the tidal turbine blade, the structural performance of the blade can be overestimated.

**Table 1:** Material properties of the UD laminates (unconditioned, 2-month conditioned and 6-month conditioned)

Property	Standard	Results			Degradation	
		0 month	2 months	6 months	2 months	6 months
0° tensile strength [MPa]	ISO 527	782 [35.94]	675 [57.79]	511 [25.88]	− 14%	− 35%
0° tensile modulus [GPa]	ISO 527	39.7 [1.50]	38 [1.08]	36.7 [1.13]	− 4%	− 8%
90° tensile strength [MPa]	ISO 527	46.5 [2.24]	51 [2.6]	43.1 [2.60]	10%	− 7%
90° tensile modulus [GPa]	ISO 527	11.9 [0.21]	11.4 [0.49]	10.4 [0.49]	− 4%	− 13%
0° compression strength [MPa]	ASTM D6641	643 [10.64]	576 [34.61]	567 [38.56]	− 10%	− 12%
0° compression modulus [GPa]	ASTM D6641	37.9 [0.71]	39.2 [0.95]	38.5 [0.68]	3%	2%
90° compression strength [MPa]	ASTM D6641	185 [9.93]	163 [6.88]	165 [8.67]	− 12%	− 11%
90° compression modulus [GPa]	ASTM D6641	14 [0.50]	13.6 [0.34]	13.7 [0.44]	− 3%	− 2%
Inter-laminar shear strength [MPa]	ISO 14130	73.9 [1.83]	–	–	–	–
In-plane shear strength [MPa]	ISO 14129	53.7 [0.33]	46.7 [1.71]	46.1 [0.65]	− 13%	− 14%
In-plane shear modulus [GPa]	ISO 14129	3.67 [0.04]	3.5 [0.18]	3.5 [0.02]	− 5%	− 5%

**Table 2:** Material properties of the BX laminates (unconditioned)

Property	Standard	Results
0° tensile strength [MPa]	ISO 527	478.1 [30.59]
0° tensile modulus [GPa]	ISO 527	25.7 [0.67]
0° compression strength [MPa]	ASTM D6641	532.7 [19.98]
0° compression modulus [GPa]	ASTM D6641	26.3 [0.64]
Inter-laminar shear strength [MPa]	ISO 14130	67.3 [1.69]
In-plane shear strength [MPa]	ISO 14129	56.8 [1.57]
In-plane shear modulus [GPa]	ISO 14129	3.7 [0.096]

**Fig. 2:**
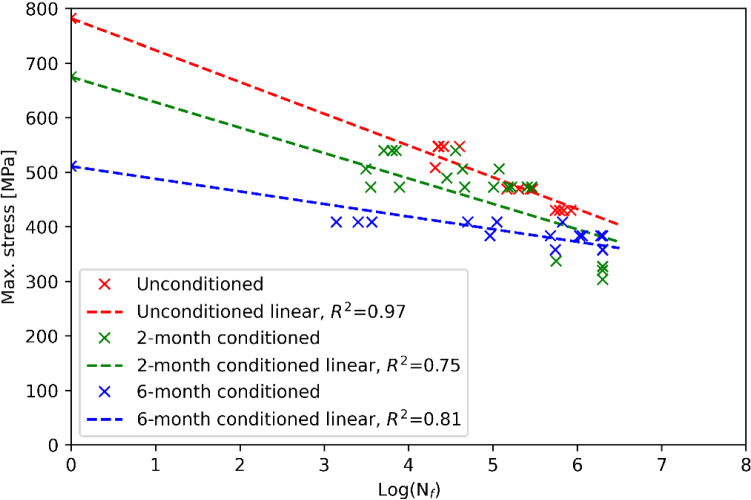
The fatigue test results of UD materials

Besides the tensile tests, fatigue tests were also carried out to characterise the resistance of conditioned and unconditioned UD laminates to failure under the repeated application of load. The loads were applied along the fibre direction of the laminates (in 0 degrees). The R-value, which is the ratio between the minimum stress and the maximum stress, was kept at 0.1. The tests were performed in accordance with the ASTM D3479/D3479M-19 (2018). Figure 2 shows the *S*–*N* curves of the unconditioned, 2-month conditioned and 6-month conditioned UD laminates. With the increase of the conditioning time, both the fatigue resistance and the slope of the *S*–*N* curve decrease. The test results are in line with the findings from the research works of Kennedy et al. (2018), where a 20 ~ 25% decrease in the fatigue strength of the aged epoxy/E-glass laminate was found. It should be noted that although the coupons were saturated by artificial seawater, the tests were conducted under dry conditions. Hence, with the increase of the cycle numbers, the coupons are likely to have dried out and the material performance may be influenced (Davies 2014). This may introduce uncertainties in the material properties.

### Layup

**Fig. 3:**
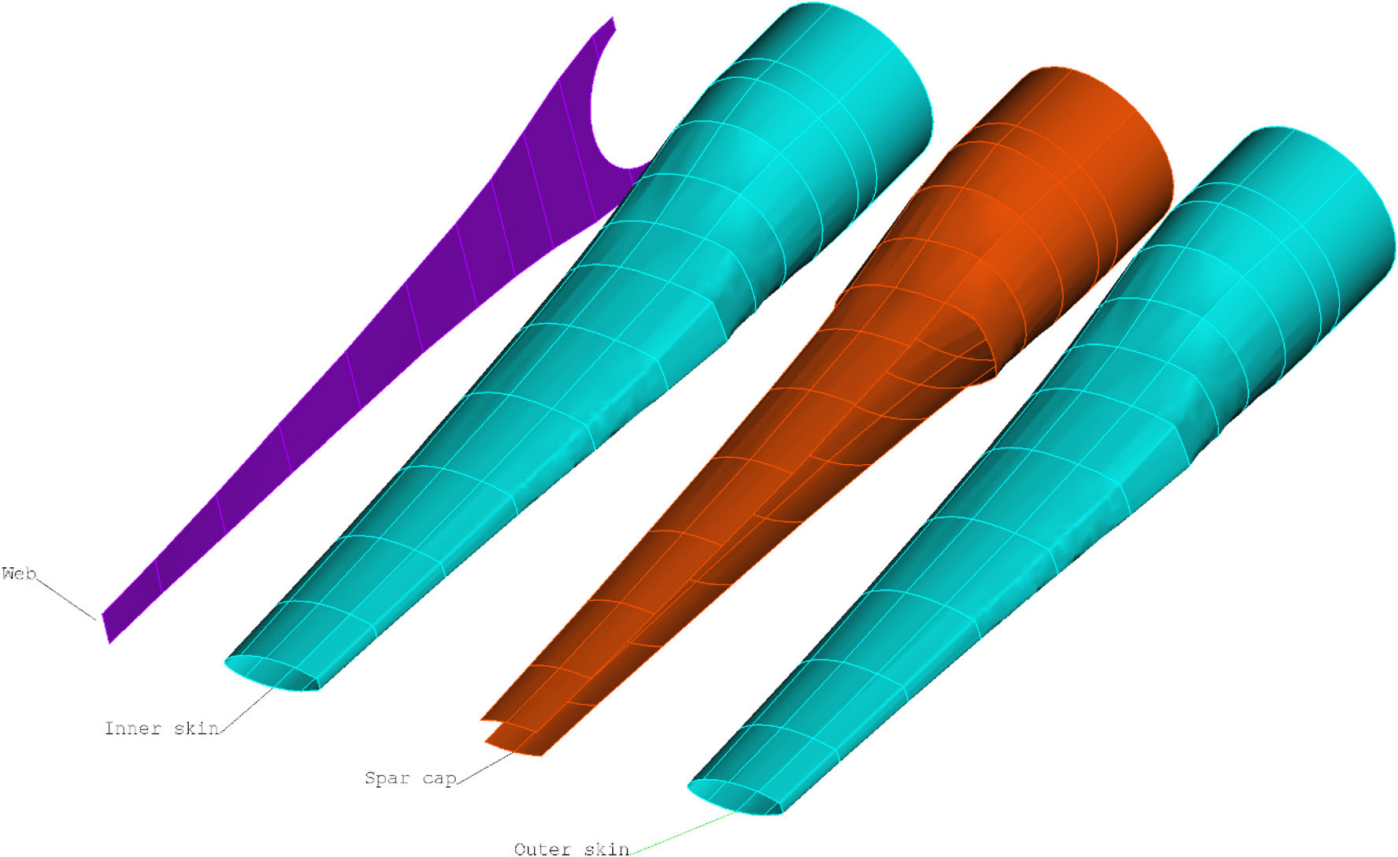
The components of the tidal turbine blade

**Fig. 4:**
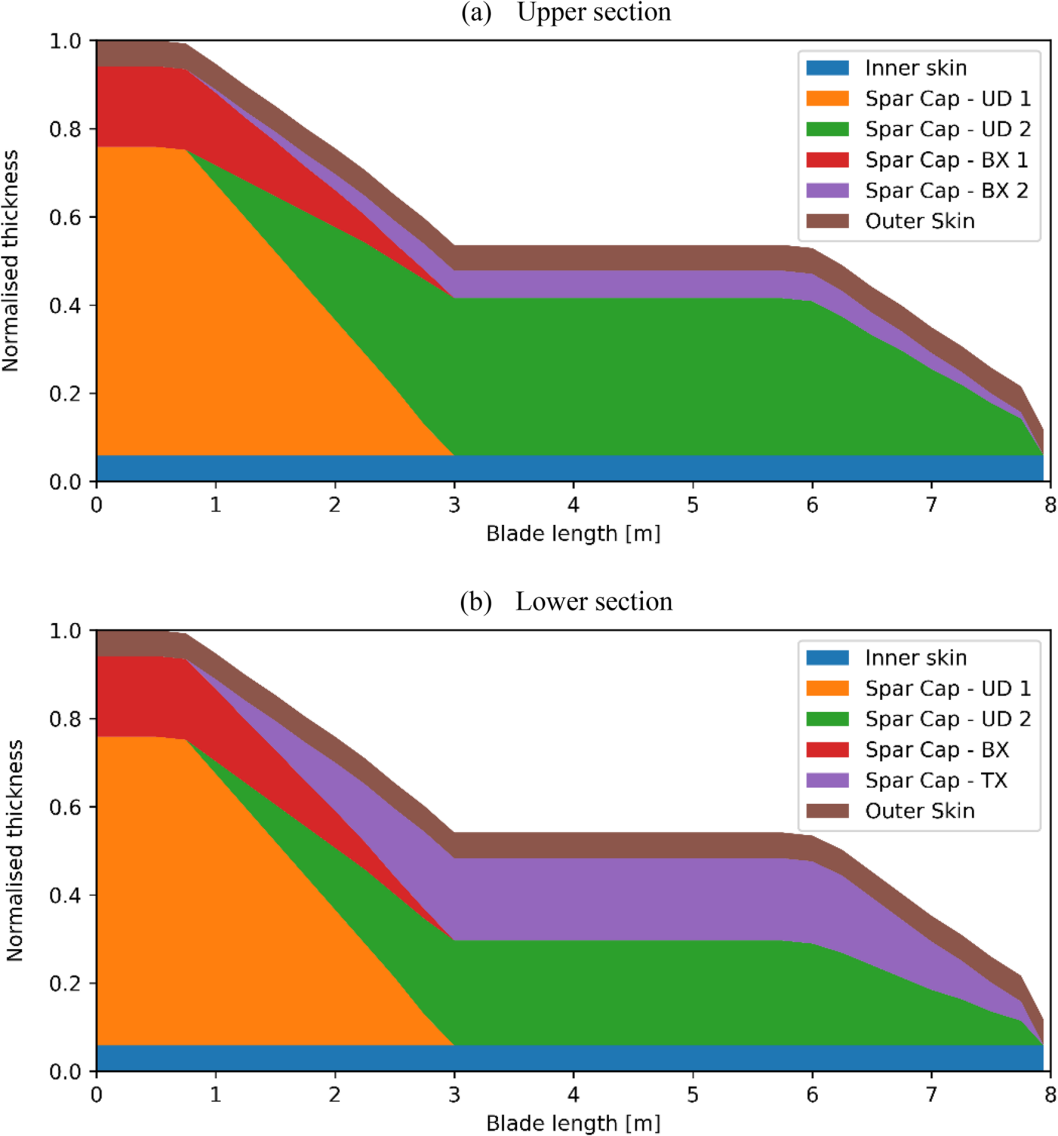
Spar cap material breakdown and thickness (including the outer and inner skin, adapted from Finnegan et al. (2021a))

According to the structural details supplied by the manufacturer, the blade can be divided into four components, namely the outer skin, the spar cap, the inner skin and the web, as shown in Fig. 3. The inner and outer skins shelter the blade from the internal and external sides. The two skins were both made with 16 layers of glass-fibre BX plies. The spar cap was manufactured using a mix of glass-fibre UD and BX powder epoxy layers. Together with the inner and outer skins, this region forms a sandwich composite component which aims to resist the hydrodynamic loads caused by the tidal current. Figure 4 shows the material details and normalised thickness of the spar cap. The blade root region is the thickest part of the blade as it should withstand the moments generated by the tidal stream. The root cross-section is designed to be circular and is connected to the turbine pitch bearing via 48 steel inserts (Finnegan et al. 2021a). Together with the inner and outer skins, the root becomes the thickest part of the blade, with a thickness of 127 mm. There is one web inside the blade, ranging from circa 1 m from the root up to the tip. The web was made with 80 layers of glass-fibre BX plies. It connects the upper and lower sides of the spar cap to form an I-beam, which enhances the strength of the spar cap. It should be noted that with only the GFRP material used, the shell of this tidal turbine blade is thick enough to avoid local buckling failure. Hence, different from a typical wind turbine blade, there are no lightweight in-fill materials used in the blade to increase the shell thickness.

### Experimental testing

**Fig. 5:**
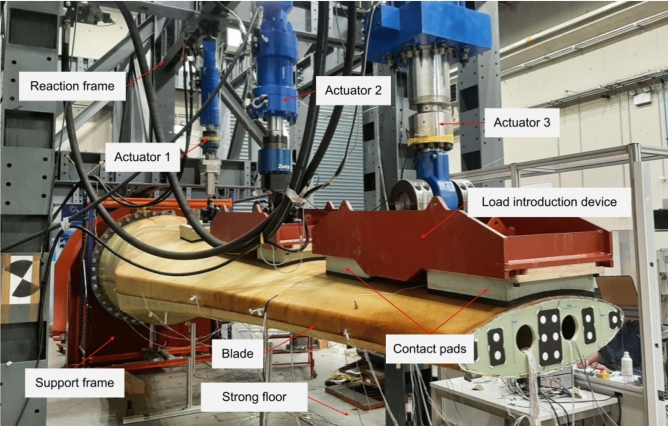
Test setup (in large structures testing laboratory at University of Galway)

A series of full-scale blade tests, aiming to study the structural performance of the tidal turbine blade, were carried out at the Large Structures Testing Laboratory at University of Galway (Finnegan et al. 2022). In this paper, the natural frequency tests, the static tests and the fatigue tests are detailed. As can be seen in Fig. 5, a steel support frame was designed and manufactured to hold the blade during static testing. The blade was mounted to the support frame at a pitch angle of 6° through 48 steel inserts, with its pressure side facing the upward direction. The support frame was fixed to the reinforced concrete reaction floor.

Natural frequency tests were performed to determine the dynamic properties of the blade. In these tests, five and three single-axis accelerometers were installed along the pressure side and the leading edge, respectively, to record the blade vibration responses. To excite the blade, a hammer was used to introduce a transient impact to the tip. The first two flapwise and edgewise modes, as well as the first torsional mode, are of interest. To obtain the flapwise and torsional modes, the transient impact was imposed in the flapwise direction, while for the edgewise mode, the edgewise impact was imposed. The natural frequencies of the blade were acquired based on Fast Fourier transform (FFT) analyses. Each type of test was repeated three times and the average values were used.

The static testing aims to ensure that the tidal turbine rotor blade can withstand the design load cases, which were provided by the blade designer. Blade design loads are calculated from time series simulation carried out using DNV software ‘Tidal Bladed’^1^ combined with a safety factor on loads of 1.35. The simulation includes the influence of twin rotor operation, floating platform, rotor legs, drivetrain and turbine control system. Maximum design loads for the blades typically result from ‘Normal operation’ around rated power (2.5 m/s current speed) in conditions close to the cut-out wave height for the turbine. The tests were carried out based on the DNVGL-ST-0164 code (2015) and the IEC TS 61,400–23 standards (2014). To distribute the design loads along the blade length, the testing loads were applied by three servo-hydraulic actuators (Fig. 5). The load applied from actuator 1 was directly distributed to the blade surface through a contact pad. Regarding actuators 2 and 3, steel load introduction devices were used to split the loads from each of the actuators equally over two contact pads. As can be seen in Fig. 5, each device was connected to the actuator through a swivel and could split the applied load into two contact pads. The five loading points improve the test accuracy when matching the resulting shear force and bending moment profiles with those experienced using design forces, ensuring an accurate representative of the tidal loads. The contact pads were manufactured using high-density polyurethane foam and were sized to fit the geometry of the blade surface. The area of each contact pad is about 3000 cm^2^. During testing, no local damage or local deformation was observed around the loading areas, proving that the contact pad size is adequate for distributing the loads.

There were eight load cases, with loads increasing up to the maximum loads, considered in the static tests. The loads applied in each test are summarised in Table 3. It should be noted that the loads were applied in the downward direction and the blade was installed at a pitch angle of 6°. Therefore, the loads applied by the actuators can be decomposed in flapwise and edgewise directions. As the edgewise extreme loads are not considered to be critical for the blade design, the actuator loads were specified so that the flapwise design loads matched closely with the flapwise design bending moments. Under the specified test loads, about 50% of the edgewise design loads were achieved. Torsional extreme loads are not considered to be critical for the blade design and, hence, were not included in the test campaign.

**Table 3:** Static load cases and the corresponding actuator loads

	Actuator 1 [kN]	Actuator 2 [kN]	Actuator 3 [kN]
Load case 12.5%	19	61	45
Load case 25%	39	123	90
Load case 37.5%	58	184	135
Load case 50%	78	245	180
Load case 62.5%	97	306	225
Load case 75%	116	368	270
Load case 87.5%	136	429	315
Load case 100%	155	490	360

Fatigue loads are also derived from time series simulation. One full lifetime is decomposed into individual load cases representing all modes of turbine operation (normal operation, startup, stops, etc.) Each case is weighted by its expected occurrence and damage equivalent loads are calculated for the resulting full lifetime load spectra. During the fatigue tests, the actuator configuration was the same as that of the static tests. The actuators applied compression–compression loading on the pressure side of the blade. Consequently, the pressure side was under tension–tension fatigue while the suction side was under compression–compression fatigue. For each actuator, the minimum load was 10% of the maximum load. Fatigue tests were performed in two phases with fatigue loads listed in Table 4. In phase 1, the testing loads were determined from previous operational trials and were converted for laboratory testing. The actual operational load was amplified to ensure that the cycle number 200,000 under the amplified load matches an equivalent of 20 years of operation. In phase 2, the loads applied in phase 1 were amplified with a factor of 1.39 and the load cycle carried out was 100,000. This is equivalent to another 20 years of operation at factored load. Details of the two fatigue load cases were provided by the blade designer.

**Table 4:** Fatigue load cases and the corresponding actuator loads

		Actuator 1 [kN]	Actuator 2 [kN]	Actuator 3 [kN]
Phase 1 (200,000 cycles)	Min load	15	19.2	9.9
	Max load	150.1	191.9	99.2
Phase 2 (100,000 cycles)	Min load	22.4	28.6	14.8
	Max load	223.6	285.8	147.8

To monitor the response of the blade under different load cases, strain gauges and displacement transducers were installed. The displacement transducers, namely linear variable differential transformers (LVDT, linearity 0.1%) and linear potentiometers (linearity 0.1%) were installed at the locations 3.28 m, 5.13 m and 7.94 (from the root) to measure the blade deflections. At 6 cross-sections (0.5 m, 2.16 m, 3.96 m, 5.13 m, 6.16 m and 7.19 m from the root), electrical resistance strain gauges (gauge factor uncertainty, 1%) were installed at different locations to monitor the strain development on the blade surface. Additionally, there were LVDTs and strain gauges installed on the support frame to monitor the blade root rotation and to ensure that the frame had no unexpected performance.

### Digital twin

Considering the complexities of the blade geometry and the layup design, the University of Galway in-house developed software, BladeComp,^2^ was used to generate the FE model of the 1-MW tidal turbine blade. BladeComp is a software specifically developed for the design and optimisation of wind and tidal turbine blades. It was previously used in many research projects for wind turbine blade analyses (Fagan et al. 2017a, 2017b, 2018; Finnegan et al. 2021b) and tidal turbine blade analyses (Fagan et al. 2016, 2019; Jiang et al. 2019). The feasibility and accuracy of the wind turbine blade FE model generated by this software were validated in many research works (Fagan et al. 2016, 2019; Jiang et al. 2019), using the experimental testing data. However, due to a lack of testing data, the accuracy and reliability of the BladeComp for predicting the structural behaviour of full-scale tidal turbine blades were still not fully examined. Hence, it is important to validate the FE model created by BladeComp using the testing data obtained in this research. This can ensure the numerical results can represent the real structural response of the tidal turbine blade.

**Fig. 6:**
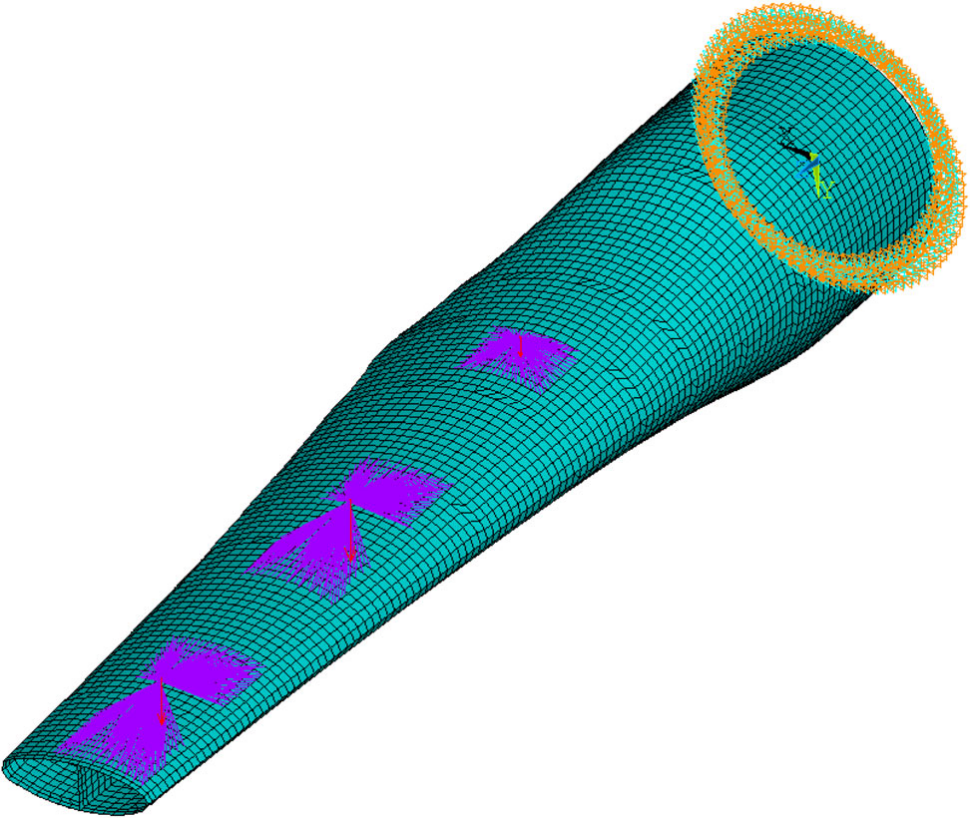
Finite-element model of the blade

Based on the blade details described in the previous subsections, the model for FE software Ansys® Academic Research Mechanical, Release 17.1 (2016) was generated by BladeComp. Similar to a typical FE model for wind turbine blades, the tidal turbine blade model is constructed with shell element SHELL281, which contains eight nodes with six degrees of freedom at each node. For each shell element, layered shell sections with multiple layers (three integration points per layer) are assigned. Figure 6 shows the meshed FE model generated for simulating the static tests. The loads applied by the three actuators were considered point loads. As shown in Fig. 6, multipoint constraint elements (MPC184) were used to distribute the point loads from the actuators to the pressure side of the blade. This mimics the load introduction mechanism of the physical tests. The constrained nodes lie in the loading areas which were covered by the contact pads. In the research works of Finnegan et al. (2021b), the same load-distributing methodology was used and its feasibility was validated. It should be noted that this modelling methodology only works when there is no slippage between the contact pad and the blade surface.

**Fig. 7:**
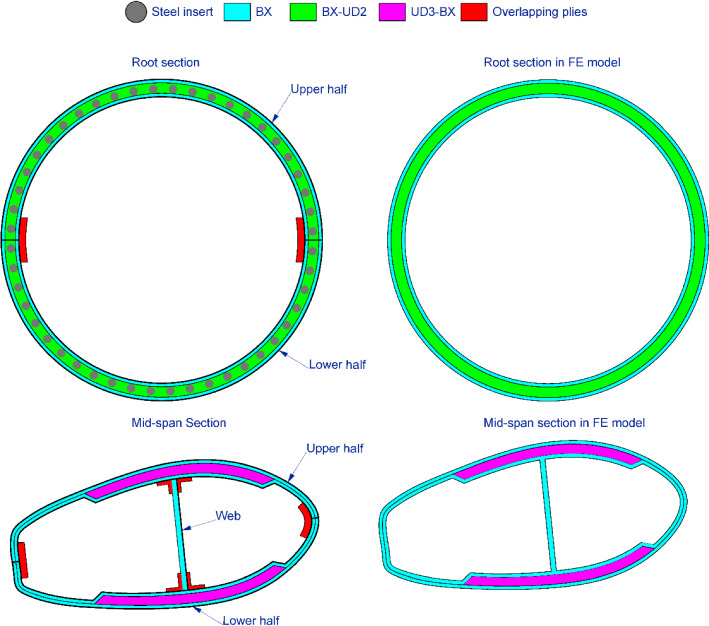
Cross-section details of the blade

It should be noted that two main simplifications were made when modelling the tidal turbine blade. The left side of Fig. 7 shows the cross-sections of the manufactured blade, while the simplified cross-sections of the FE model are shown on the right side. As detailed in Finnegan et al. (2021a), the blade consists of three parts, namely the upper half, the lower half and the web. This is because the blade was manufactured in two stages. In the first stage, the upper half, the lower half and the web of the blade were built separately using CPET. In this stage, the three components were not fully cured. In the second stage, the three components are assembled together and additional uncured overlapping plies are added to ensure that continuous fibres are running between all join lines. Finally, the entire blade is fully cured in situ under vacuum bag only at high temperatures. Hence, at the bonding surfaces, the fibre was not continuous. This fibre discontinuity is difficult to represent using shell elements. Considering that the numerical model is developed for predicting the behaviour of the full-scale blade, these local structure details can be simplified, given that no damage is observed at the bonding interfaces. Hence, these overlapping plies were neglected in the digital twin. The fibre was assumed to be continuous across different parts of the blade, as shown in the simplified cross-sections of Fig. 7. Besides that, the elements on the web are directly connected to the elements of the blade surface by sharing the same nodes. The second simplification was made on the steel inserts which were embedded at the root section during the first manufacturing stage. In the tests, the blade was fixed to the support frame through these 48 steel inserts (Finnegan et al. 2021a). However, considering that it is relatively difficult to model the embedded steel inserts in shell elements, these inserts were neglected during FE modelling. Instead, the nodes at the root sections were directly constrained. By simplifying the FE model, in terms of neglecting overlapping plies and steel inserts, the mass predicted by the FE model (3.82 tonnes) is lower than the experimental value (more than 4 tonnes). This change in mass will influence the results of the natural frequency analysis. Moreover, neglecting the steel inserts may also impact the stress/strain distribution on the root section. These will be discussed in Sect. 3.

The mechanical properties obtained from the coupon tests were used as the material input data of the FE model. However, it should be noted that the coupon test results may not perfectly represent the material performance of the blade structure, despite that the same manufacturing methodology was used. This is mainly due to the difference in the thickness (or ply number) between the coupons and the actual blade. Different thicknesses may lead to different fibre volume fractions and cure states, resulting in uncertainties in the mechanical properties. Besides that, other factors, such as mould shapes and manufacturing defects, would also influence the material properties. However, limited by the capacity of the testing machine, the coupon thickness cannot be as thick as that of the actual blade structure. Hence, the material properties of the blade materials were assumed to be the same as coupon test results in this research.

**Fig. 8:**
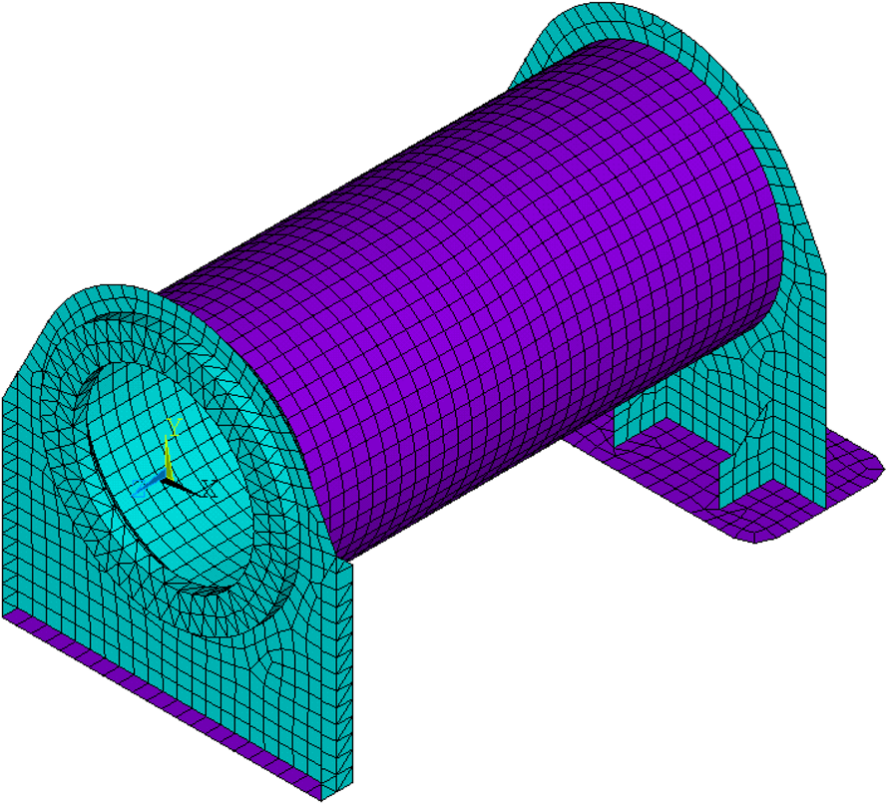
Finite-element model of the support frame

For the natural frequency analysis, the FE model for the steel support frame, shown in Fig. 8, was included as it is considered to influence the vibration properties of the testing system. The frame was modelled with a combination of solid elements (SOLID185) and shell elements (SHELL181). The blade root was directly constrained to the support frame using bonding elements.

## Results and discussion

### Digital twin validation

In this section, the feasibility and accuracy of the FE model generated by BladeComp are validated against the testing data, including the natural frequency tests and the static tests. It should be noted that the blade was tested in dry conditions so that the unconditioned material properties were used.

#### Natural frequencies

Two natural frequency analyses were performed in this study, one with the supporting frame, and one without the supporting frame. Table 5 summarises the natural frequency values obtained from testing and predicted by the FE model. The numerical results (without the supporting frame) do not have good agreements with the test data. The first three predicted frequencies are more than 40% higher than the test results, while the predicted first torsional frequency is 15% less than the test value. Additionally, the second edgewise mode was not captured by the FE analysis. When including the supporting frame in the analysis, the predicted natural frequencies become closer to the test results compared to that of the FE analysis without the support frame, with improvements ranging from 6 to 16%. This indicates that the steel frame was also vibrated under the transient impact on the blade tip, and hence, has a non-negligible influence on the testing results. However, the differences between the FE model predictions (with the supporting frame) and the experimental results are still more than 12%. The inaccurate numerical values could result from the simplifications made when generating the FE model. By neglecting the overlapping plies and steel inserts, the FE model becomes lighter in mass, and therefore, results in a higher frequency prediction. Another important aspect to influence the natural frequency result is the flexibility of the connections between the support frame and the strong floor. During the static testing, some movements were observed at these connections, indicating that the connections were not perfectly constrained. This flexibility could have an impact on the vibration of the blade with its support structure, resulting in a lower natural frequency value compared to the blade with perfect boundary conditions. The mismatch between predicted (without supporting frame) and experimentally measured natural frequencies indicates that the support frame may play an important role in the natural frequency tests. To obtain a relatively accurate result, the stiffness and self-weight of the support frame should be significantly larger than that of the blade. Another possible reason for disagreement with respect to the natural frequencies can be the uncertainty regarding the fibre fraction. As discussed in the Sect. 2.5, although the same methodology was used to manufacture the composite coupons and the blade, the mechanical properties of the coupons and blade structure can be different. This is caused by the differences in component size, thickness and mould shape of the manufactured components. This may introduce uncertainty of the material properties, hence, resulting in the difference of natural frequencies.

**Table 5:** Natural frequencies from the test and the FE analysis

Mode	Test results [Hz]	FE results [Hz] (with supporting frame)	FE results [Hz] (without supporting frame)
1st Flapwise	15.28	17.98	21.29
1st Edgewise	17.81	19.98	23.26
2nd Flapwise	31.34	45.11	48.16
2nd Edgewise	31.48	54.27	NA
1st Torsional	84.51	65.02	57.14

#### Static testing

**Fig. 9:**
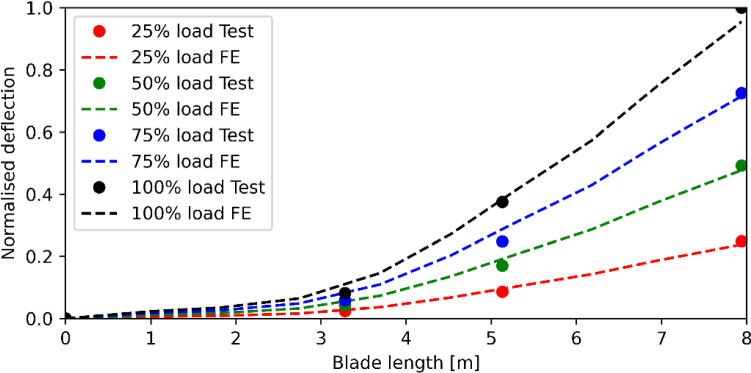
Normalised deflection comparisons between the testing data and FE results (normalised to the maximum deflection at 100% load case)

With the increase of the loads in the eight load cases, the blade was observed to behave consistently throughout the static testing. Hence, four load cases, namely 25%, 50%, 75% and 100% of design load cases, were selected for FE model validations, to reduce congestion in displaying the results. In the physical tests, no failure or cracks were observed under the maximum design load (100% load case), demonstrating that the blade has the structural integrity to withstand the design loads. Under the static loading, the blade root slightly rotated under the large root moment (5714 kN·m). This root rotation was obtained by the displacement data from a pair of LVDTs installed at the support frame. Under the 100% load case, a root rotation of 0.17° was observed. Considering that the blade is about 8 m long, this rotation resulted in an additional deflection of circa 23 mm at the tip. Hence, the deflection values recorded by the three displacement transducers were corrected with the root rotation angles. With the root rotation corrected, the supporting frame can be neglected in the static FE analyses. Figure 9 summarises the blade deflections under the four load cases, where the deflections are normalised according to the ultimate value in the plot. The maximum blade tip deflection is about 1.3% of the blade length, indicating that the blade will not hit other components of the tidal turbine under extreme conditions. Moreover, considering that the tidal force is smaller compared to the extreme load case, the blade can maintain a good shape during operation, ensuring normal energy capturing by the turbine.

The predicted blade deflections from the FE model are compared with the test data in Fig. 9. In the four load cases, the FE model is observed to overestimate the blade deflections at the locations 3.28 m and 5.13 m and slightly underestimate the blade deflection at the tip. There are two reasons which can lead to stiffness underestimation in the root region. Firstly, as discussed in the previous section, the steel inserts contribute stiffness to the root region. Secondly, wrinkles, formed when placing the plies on the curved mould, were observed in the internal surface of the blade. Thick laminates, especially around the root region, are likely to develop significant wrinkles during manufacture, which may have an impact on the root region stiffness. However, in general, the numerical results from the FE model have very good agreement with the experimental data. Under the 100% design load, the predicted tip deflection was about 100 mm, which is within a 10% difference from the value obtained from the physical laboratory test. Hence, the FE model could predict the stiffness of the tidal turbine rotor blade accurately.

**Fig. 10:**
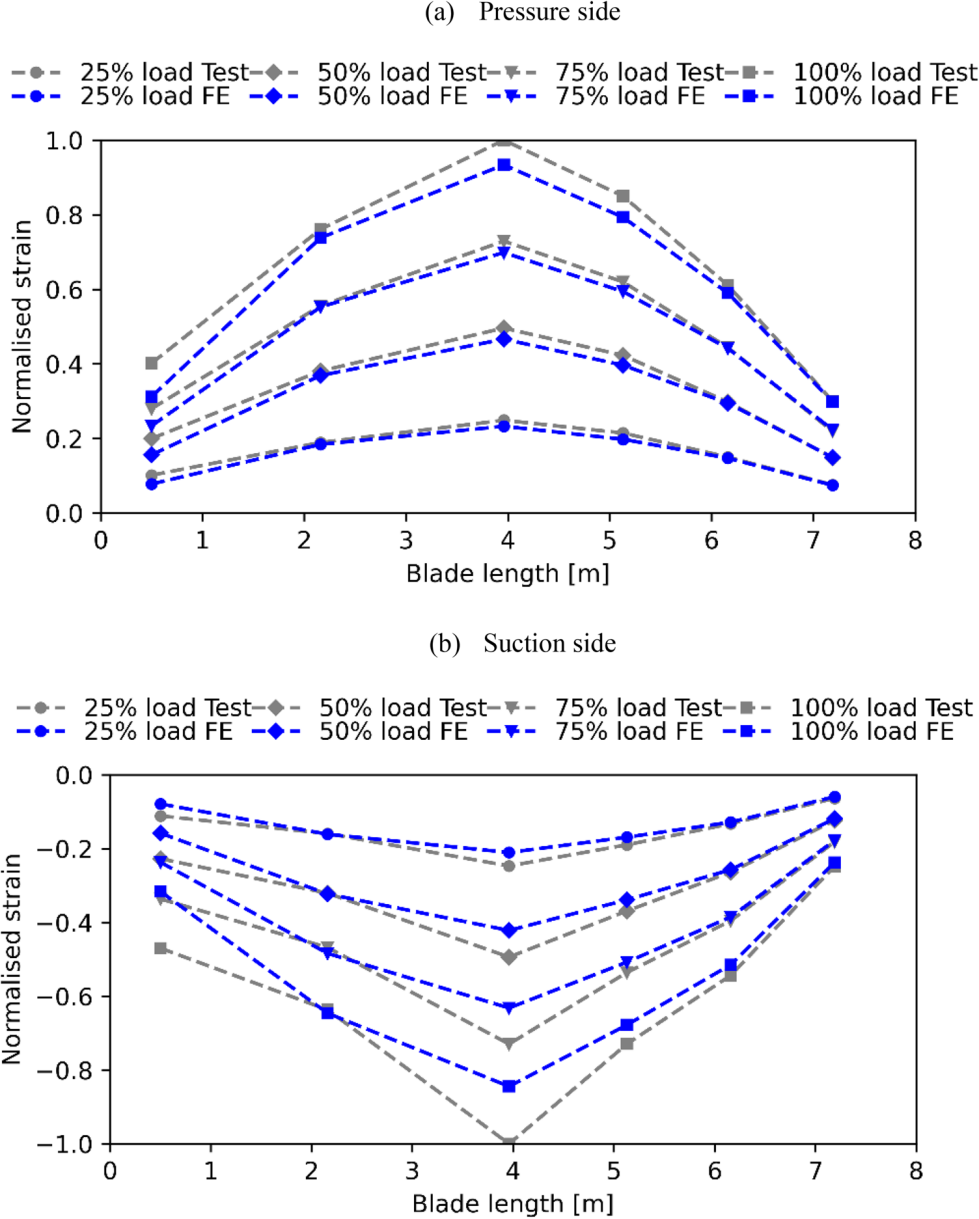
Normalised blade longitudinal direction strain comparisons along the length of the blade between the testing data and FE results

Under the testing loadings, the pressure side and the suction side were observed to suffer the highest tensile strain and compressive strain, respectively. The normalised longitudinal direction strain along the pressure side and suction side of the selected four load cases are plotted in Fig. 10. The longitudinal direction is defined as the reference direction used for laying up the composites during manufacturing, which is from the root to the tip. The maximum absolute strain, 0.002, occurred on the pressure side of the spar cap, under the 100% load case. The strain limit provided by the turbine designer is about ± 0.025, which is also in line with the design guidelines provided in DNVGL-ST-0164 (2015), indicating that the blade meets the design expectations. The strain data predicted by the FE analyses are plotted and compared with the test data in Fig. 10. The predicted strain values around the blade root are lower than those from the test results. This contradicts the observations in the deflection comparisons, where the stiffness of the blade around the root region was underestimated by the FE model. However, considering that the strain gauge just measures the strain value of a single location in the blade surface, the strain recordings of the two gauges could not indicate the strain distribution of the root region. Hence, it is difficult to identify if the stiffness around the root region is underestimated or not from the few strain values measured. Another potential reason which may cause the mismatch is the simplification made when generating the FE model. By neglecting the steel inserted embedded at the root section, the stress and strain distributions around the root can be impacted. This is in line with the observation in the research work carried out by Finnegan et al. (2021b). Overall, strain values predicted by the FE model strain show good agreement with those from the strain gauges. Most of the predicted values are marginally lower than the test data. It indicates that the FE model has slightly overestimated the blade stiffness, which is in line with the observations from the tip deflection comparisons.

### Influence of seawater ingress

Considering the tests were conducted in dry condition, the impact of seawater ingress on the composite materials were not considered in the static tests. As discussed in Sect. 2.2, the material conditioned in seawater has a considerable strength degradation, indicating that the blade responses under the testing loads can be conservative. Given that the FE model generated by BladeComp can accurately predict the structural behaviour of the tidal turbine blade, numerical analyses were conducted to study the influence of seawater on the blade behaviour under extreme loads. In the numerical analyses, the material properties of the conditioned composite coupons were used as the input data. However, it should be noted that the mechanical properties of the conditioned coupons may not perfectly represent the material properties of the seawater-immersed blade. Regardless of uncertainties introduced during manufacturing, the thickness of the composites plays an important role on the seawater ageing effects. Thicker laminates have better resistance to seawater ageing effects compared to that of thinner laminates. However, considering that the conditioned coupons are thinner than the blade structure, the predicted results are conservative and ensure that the analysis stay on the safe side. It should be noted that the inter-laminar shear strength of the conditioned glass-fibre UD laminate was not tested. Hence, the degradation of the in-plane shear strength of glass-fibre UD composite was applied to the inter-laminar shear strength. This assumption was made due to a lack of input data, which can introduce uncertainties to the analysis results. Moreover, only unconditioned BX glass-fibre laminates were tested in this research. It is assumed that the UD and BX materials share the same degradation levels under the seawater ingress. The impact of material strength degradation on the blade deflection, blade ultimate strain and blade failure is studied in this section. The 100% load case was considered in the analyses as it represents the most critical load condition.

**Fig. 11:**
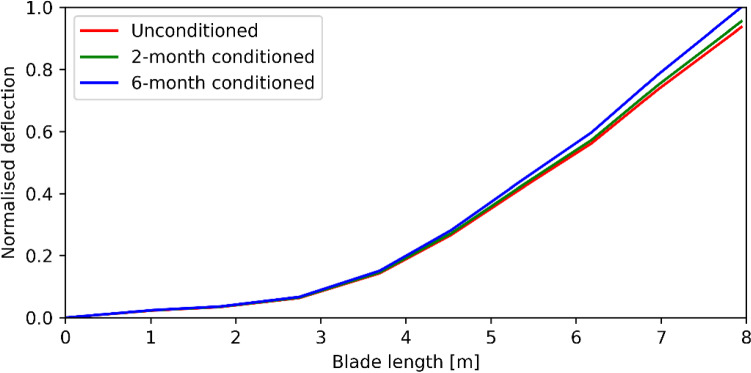
Blade deflection comparisons under different material degradations (normalised by the ultimate tip deflection)

The normalised blade deflection under the 100% design loads, with two material degradation conditions, are plotted in Fig. 11. The blade stiffness was negatively impacted by the seawater ingress. The blade tip deflection has an increase of 2% and 7% under 2-month conditioned and 6-month conditioned, respectively. It indicates that the testing results stay on the unconservative side.

**Fig. 12:**
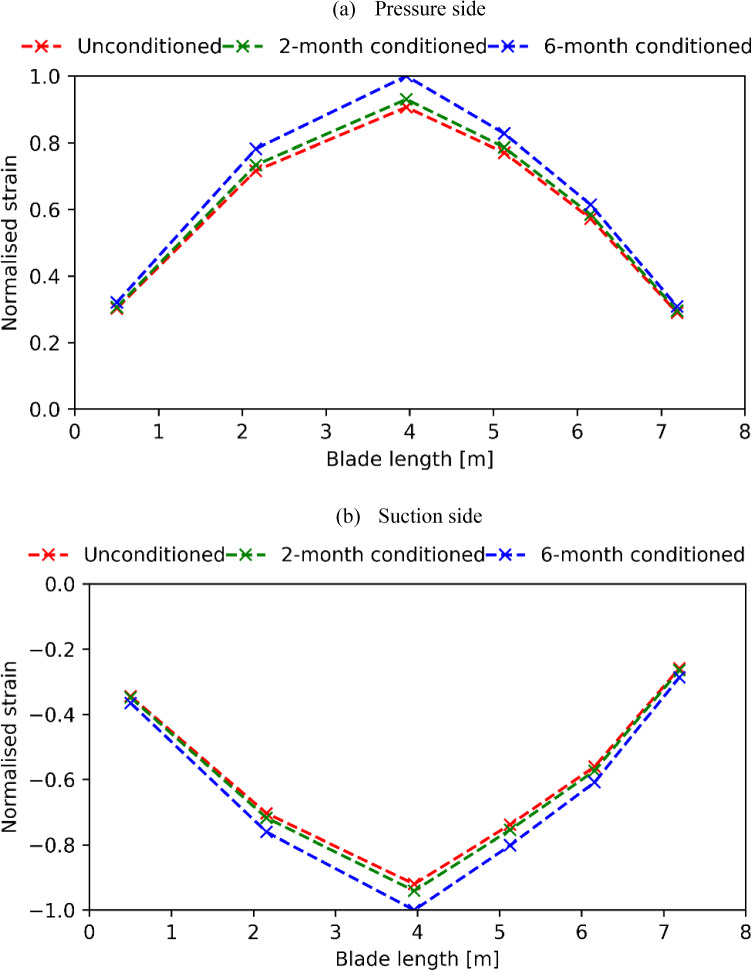
Normalised blade longitudinal direction strain comparisons under different material degradations

The normalised strain distribution on the pressure side and suction side of the blade are plotted in Fig. 12. Similar to the observation from the deflection comparisons, seawater has a negative impact on the strain of the blade. The strain on both sides of the blade has increased by 1 to 3% under 2-month conditioning, while increases of 6 to 11% are observed under the 6-month conditioning.

With the stress provided by the FE model, the failure analysis, based on the Puck failure theory (Puck and Schürmann 1998), was performed to evaluate the risk of composite failure under the 100% design loads. The Puck failure theory classifies the failure mode of composite laminate into fibre failure and matrix failure, with the corresponding maximum inverse reserve factors listed in Table 6. The inverse reserve factor represents the risk of failure calculated based on the Puck failure theory. If the inverse reserve factor of an element on the blade is larger than 1, this element suffers a composite failure. Under the seawater ingress, the inverse reserve factor for the matrix failure increases by about 19% under both 2-month and 6-month conditioning. Regarding the fibre failure, it is found that the maximum inverse reserve factor of the blade has an increase of 12% and 39% under the 2-month and 6-month conditioning, respectively. This is in line with observation in the material testing, where the fibre direction strength of the UD was found to have the highest strength degradation (about 35%). However, all the inverse reserve factors are less than 1, indicating the current blade structural design is safe under static loading, with the consideration of seawater impact. It should be noted that these analyses did not consider the risk of interlaminate failure since the shell elements utilise the plane-stress assumption and ignore the out-of-plane stress. According to the research works of Harper et al. (2016), tidal turbine blades have risks of inter-laminar crack growth and delamination at ply drops, where out-of-plane stresses can play an important role.

**Table 6:** Maximum failure inverse reserve factors under different material degradations

	Fibre	Matrix
Unconditioned	0.18	0.72
2-month conditioned	0.20	0.85
6-month conditioned	0.25	0.86

From the aforementioned observations, the structural performance could be overestimated if the seawater impact is neglected. Hence, for testing a tidal turbine blade, it is recommended to amplify the testing loads to consider the seawater impact. By carrying out several trial analyses, it is found that to apply amplification factors of 7% and 10%, the blade ultimate deflection and ultimate strain match well with that of the testing data, respectively. Hence, for this tidal turbine blade, a 10% amplification factor is suggested to take into account the seawater immersion impact in a conservative way.

The fatigue damages of the tidal turbine blade under the different seawater ingress conditions were analysed based on the stress outputs from the FE analyses. The fatigue damages along the fibre direction and normal to the fibre direction of the UD laminates were analysed. For the fatigue damage along the fibre direction, the *S*–*N* curves from the fatigue coupon tests were used. As the fatigue property normal to the fibre direction of the unconditioned UD material was not tested, data from the OptiDAT materials fatigue database (Nijssen et al. 2006) was used. For the conditioned materials, the slopes of the *S*–*N* curves were assumed to be the same as that of the unconditioned materials. As observed from Fig. 2, the slope of the *S*–*N* curve of unconditioned material is steeper than that of conditioned material. It indicates that applying this assumption can overestimate the fatigue damage, making the analysis stay on the safe side. Miner’s rule (1945) was used to calculate the fatigue damage, which is summarised in Table 7. It should be highlighted that strength degradation of the composite material under the fatigue load was not considered in this research. Based on the analysis results, the maximum fibre direction stress is less than 30 MPa among the three conditions, which is not expected to cause significant fatigue damage to the material. Hence, the fatigue fractions along the fibre direction under the three conditions can be considered as 0 after the two phases of fatigue cycles. Regarding the fatigue damage normal to the fibre direction, it is significantly higher than that along the fibre direction. The damage fraction increases with the increase of conditioning time. However, the maximum damage fraction is less than 0.01 (fatigue damage occurs when the damage fraction is larger than 1). This indicates that fatigue damage introduced to the blade after the two phases of tests can be considered to be mild. This is in line with the observations from the fatigue tests. Based on the natural frequency tests and residual strength tests performed after the fatigue tests, no stiffness degradation and natural frequency change were observed. Hence, even if the material suffers fatigue resistance loss under seawater impacts, the fatigue life of the blade is not influenced. This is benefitted from the high strength of the blade, indicating that the blade may be conservatively designed.

**Table 7:** Fatigue analysis results under different material saturation conditions

Saturation conditions	Fatigue damage along the fibre direction			Fatigue damage normal to the fibre direction		
	Cycles to failure (Phase 1)	Cycles to failure (Phase 2)	Damage fraction	Cycles to failure (Phase 1)	Cycles to failure (Phase 2)	Damage fraction
Unconditioned	1.27E + 13	8.82E + 12	≈ 0	5.11E + 08	2.08E + 08	8.72E−04
2-month conditioned	1.18E + 14	7.43E + 13	≈ 0	4.13E + 09	1.66E + 09	1.09E−04
6-month conditioned	1.85E + 21	6.97E + 20	≈ 0	9.39E + 07	3.65E + 07	4.87E−03

## Conclusions

In this paper, a series of studies, including experimental tests and numerical analyses, were carried out to study the structural behaviour of a 1 MW tidal turbine rotor blade under dry and seawater ingress conditions. Composite coupons, conditioned using artificial seawater, were prepared and tested to study the impact of seawater on the material strength. According to the blade design details and the testing programme, a digital twin in the form of an FE model, constructed by the in-house developed software, BladeComp, was created for the tidal turbine rotor blade. With the accuracy of the FE model validated against the physical testing results, numerical analyses were conducted to study the performance of the tidal turbine blade under the design loads. The impact of the seawater ingress on the testing results was investigated and discussed. The following conclusions are proposed:

- Conditioned with artificial seawater, the composite coupon loses its strength considerably. The most significant degradation occurred in the fibre direction, with a decrease of 35% observed from the tensile coupon tests.
- The designed steel support structure may have a significant influence on the blade dynamic properties and, therefore, cannot be neglected.
- By performing static testing, the tidal turbine blade was proved to withstand the maximum design loads. No failure or cracks were observed during testing.
- The FE model generated by the BladeComp software can give an accurate prediction of the blade deflection under testing loads. Moreover, the strain values on the spar cap can also be accurately forecasted, indicating that the BladeComp is suitable for modelling tidal turbine blades.
- The seawater was found to have significant negative impacts on the blade strength, in terms of blade stiffness and risk of composites failure and, therefore, cannot be ignored. The experimental tests carried out in dry conditions could overestimate the structural performance of the tidal turbine blade.
- The fatigue resistance of the composite material is found to be impacted by the seawater ingress. However, due to the conservative structural design of the blade, the damage caused by the imposed fatigue cycles is found to be negligible, even under the 6-month saturated condition.

It can be concluded that the laboratory tests can give a preliminary estimation on the performance of a tidal turbine blade. But the results are still on the unconservative side. In the future, operation trials in sea sites are suggested. By monitoring the real-time strain data, a better understanding of the blade performance in operating conditions can be achieved and the digital twin can be further validated. It is also suggested to saturate a full-scale blade in seawater prior to the laboratory mechanical testing of the full-scale blade in the future to investigate if seawater ingress has any impact on its performance.

## Footnotes

1 Tidal turbine software modelling tool | TidalBladed. https://www.dnv.com/services/industry-standard-tidal-turbine-software-modelling-tool-tidalbladed-3799. Accessed 04 Apr 2022.

2 BladeComp – University of Galway. https://www.nuigalway.ie/structures/bladecomp/#. Accessed 10 Apr 2022.
